# Robustness of Food Processing Classification Systems

**DOI:** 10.3390/nu11061344

**Published:** 2019-06-14

**Authors:** Rachel Bleiweiss-Sande, Kenneth Chui, E. Whitney Evans, Jeanne Goldberg, Sarah Amin, Jennifer Sacheck

**Affiliations:** 1 Friedman School of Nutrition Science and Policy, Tufts University, Boston, MA 02111, USA; jeanne.goldberg@tufts.edu; 2 Tufts University School of Medicine, Tufts University, Boston, MA 02111, USA; kenneth.chui@tufts.edu; 3 Warren Alpert Medical School, Brown University, Providence, RI 02912, USA; whitney_evans@brown.edu; 4 Department of Nutrition and Food Sciences, University of Rhode Island, Kingston, RI 02881, USA; sarah_amin@uri.edu; 5 Milken Institute School of Public Health, The George Washington University, Washington, DC 20037, USA; jsacheck25@gwu.edu

**Keywords:** children, diet, processing, classification, nova, ultra-processed, micronutrients

## Abstract

Discrepancies exist among food processing classification systems and in the relationship between processed food intake and dietary quality of children. This study compared inter-rater reliability, food processing category, and the relationship between processing category and nutrient concentration among three systems (Nova, International Food Information Council (IFIC), and University of North Carolina at Chapel Hill (UNC)). Processing categories for the top 100 most commonly consumed foods children consume (NHANES 2013–2014) were independently coded and compared using Spearman’s rank correlation coefficient. Relative ability of nutrient concentration to predict processing category was investigated using linear discriminant analysis and multinomial logistic regression and compared between systems using Cohen’s kappa coefficient. UNC had the highest inter-rater reliability (*ρ* = 0.97), followed by IFIC (*ρ* = 0.78) and Nova (*ρ* = 0.76). UNC and Nova had the highest agreement (80%). Lower potassium was predictive of IFIC’s classification of foods as moderately compared to minimally processed (*p* = 0.01); lower vitamin D was predictive of UNC’s classification of foods as highly compared to minimally processed (*p* = 0.04). Sodium and added sugars were predictive of all systems’ classification of highly compared to minimally processed foods (*p* < 0.05). Current classification systems may not sufficiently identify foods with high nutrient quality commonly consumed by children in the U.S.

## 1. Introduction

The prevalence of childhood overweight and obesity remains elevated in the United States (U.S.), particularly in lower-income and minority populations [1,2,3,4]. It is widely accepted that poor diet is a key contributor to caloric imbalances and weight status in children. Over the past decades, researchers have documented eating pattern shifts that favor sweetened beverages over water and milk [5], takeaway food over meals eaten at home [6], and snacking over traditional meal patterns [7], collectively referred to as the nutrition transition [8]. Modern industrial food processing is a common denominator driving these dietary shifts. Accordingly, processed foods have been advanced as a potential driver of the child obesity epidemic, but their role in terms of nutrition and health in modern day diets is currently under debate [9].

Food processing, in forms such as cooking, fermenting, and salt-preservation, has been an integral part of human diets for over two-million years [10,11]. However, modern food processing techniques, which were introduced during the industrial revolution and take place on a mass scale, are relatively new [10]. The U.S. Department of Agriculture (USDA) defines food processing as any procedure that alters food from its natural state [12]. Thus, any food aside from raw, agricultural commodities is considered processed. Because of the considerable heterogeneity across industrially processed foods, researchers have proposed frameworks that aim to classify foods according to the category or complexity of processing, ranging from minimally to highly processed [13,14,15]. 

Several systems have been applied to dietary data collected in the U.S. or globally. These include the Nova system, developed in Brazil and used internationally in research [14]; a system developed by the International Food Information Council (IFIC) and used to examine the nutrient quality of foods consumed by Americans by processing category [15,16], and one by researchers at the University of North Carolina at Chapel Hill (UNC) that categorizes all barcoded foods items sold in U.S. supermarkets [13]. 

Several key differences between these systems are readily apparent. The Nova system divides foods into four categories [14], IFIC into five categories [17], and UNC into seven categories of processing [13] (Table 1). Nova and UNC define the lowest processing categories as “unprocessed and minimally processed” while IFIC uses the term “minimally processed.” Descriptors of the highest processing category are also varied. Nova uses the term “ultra-processed”, IFIC defines a category called “prepared foods/meals”, and UNC specifies a group called “highly processed stand-alone.” Notably, papers describing the Nova and IFIC systems include technical definitions of each processing category and a short list of example foods, while the UNC system includes an extensive list of foods that may be purchased in U.S. supermarkets by processing category, reducing subjectivity [13]. The authors of Nova have released multiple versions of the system, including ones that range from three to five processing categories, with variable definitions and example foods [18]. 

Discrepancies between these classification systems have resulted in varying conclusions regarding the relationship between processed food consumption and dietary quality. For example, an analysis of NHANES 2009–2010 data using the Nova system found that added sugar in foods from the highest processing category (ultra-processed) was eightfold higher than foods in the next highest category (processed) [19]. Similarly, a study using the UNC system found that households were significantly more likely to exceed maximum daily recommendations of saturated fat, sugar, and sodium content with highly compared to less-processed food purchases [13]. In contrast, a study using the IFIC system demonstrated that processing is a minor determinant of nutrient contributions in the diet [15].

Proponents of processing frameworks argue that traditional food classifications, such as nutrient density and food groups, fail to distinguish between unaltered and reformulated versions of a food, potentially leading to misclassification of healthful products [20]. In addition, by translating nutrient-based targets into food-based recommendations, dietary guidelines may encourage extreme levels of fortification by the food industry to portray highly processed foods as nutritious [21]. However, without established definitions of processing categories and consistent group assignment, it is impossible to compare results across studies. A direct comparison of these classification systems is necessary to avoid further misunderstandings and potential misclassification of foods in future research [22]. 

The specific aim of this study was to evaluate the robustness of processing classification systems and to assess their utility as a measure of healthfulness in children’s diets. The objectives of this study were to (1) investigate the inter-rater reliability of three food processing classification systems, (2) compare classification agreement between the three systems using the top 100 most commonly consumed foods among children in the U.S., and (3) determine whether nutrient concentrations were predictive of each system’s processing categorization for the top 100 foods. Our overarching hypothesis was that conclusions regarding the relationship between processing category and nutrient concentration will vary depending on the processing classification framework used in analyses. Specifically, we hypothesized that the UNC system would have the highest inter-rater reliability; the Nova system would be more likely to classify foods as ultra or highly processed compared to IFIC or UNC, while IFIC would classify the least foods as highly processed, and that overconsumed nutrients would be more predictive of higher processing category using the Nova classification system as compared to IFIC and UNC.

## 2. Materials and Methods 

### 2.1. Sample Size and Data Source 

Since an overarching aim of this research was to evaluate whether processing category is a useful measure of healthfulness in children’s diets, we determined the 100 most commonly foods consumed by children, ages six to twelve years old, who participated in the National Health and Nutrition Examination Survey (NHANES) 2013–2014 [23]. For validation or reliability studies estimating agreement between two different variables, a sample size of 100 gives very good precision, while 60–70 observations is generally accepted as adequate [24]. All participants in the 2013–2014 cycle were asked to complete two 24-h recall dietary interviews, conducted using the USDA Multiple-Pass Method (AMPM) by trained interviewers [25]. For children under nine years old, the interview was conducted with a proxy who was knowledgeable about the child’s consumption the day before the interview. For children 9–11 years old, the child provided their own data with an adult household member present [25]. For this study, we analyzed food records from children who completed both days of dietary recalls and had no missing demographic data. 

The top 100 foods were identified according to total reported servings consumed by children ages six to twelve years. Servings, rather than count, volume, or weight, was chosen to avoid biasing the sample toward foods eaten often but in small servings, low-weight foods with added volume (such as puffed snacks) and very heavy foods (beverages and soups). To determine the total number of servings consumed for each food item, NHANES food records from children participating in the 2013–2014 cycle were merged with the Food and Nutrient Database for Dietary Studies (FNDDS) 2013–3014 portions and weights database by Standard Reference (SR) code [26]. The FNDDS is used to convert foods and beverages from NHANES into portion weights, and to determine their nutrient values [26]. The FNDDS database contains multiple portion sizes and associated gram weights for each food (for example, one tablespoon of cereal, half a cup of cereal, one container of cereal). To ensure consistency, portions were chosen based on the standard serving size published in the United States Department of Agriculture (USDA) Nutrient Reference Database for individual items [27]. Once a portion size and associated weight was established for each item, the total number of portions was calculated by dividing the gram weight consumed by the portion size weight. The resulting food list was organized by total servings and the top 100 foods were used in subsequent analyses.

To allow for an analysis based on the nutrient content of foods according to their processing category, the most commonly under- and overconsumed nutrients in the U.S. were identified according to the Dietary Guidelines for Americans (DGA) [28]. Under-consumed nutrients include potassium, fiber, choline, magnesium, calcium, iron, and vitamins A, D, E, and C. Overconsumed nutrients include added sugars, saturated fat, and sodium [28]. Association between energy content and processing category was also explored. For this analysis, we determined nutrient values for 100 grams of each food using the FNDDS nutrient values database [27]. Amount of added sugars was obtained from the Food Patterns Equivalents Database 2013–2014 [27]. 

### 2.2. Processing Classification Systems 

Food processing classification systems applied to North American food purchase or consumption datasets, referenced in published scientific literature, were used in analyses. Three systems meet these criteria, based on a systematic review by Moubarac et al. [29] and further literature review by the authors. The systems include Nova, developed by researchers at the Centre for Epidemiological Studies in Health and Nutrition at the School of Public Health, University of São Paulo [14]; a system developed by researchers at UNC for barcoded food items in the U.S. [13], and a system devised by the IFIC [17]. The UNC system is based on the Nova system, but modified to capture the complexity of the U.S. food supply with enhanced category definitions and examples [13]. Details of each system are presented in Table 1. 

### 2.3. Processing Classification Category Assignment 

Two PhD-level registered dieticians (authors 3 and 5) independently coded the top 100 foods by processing category using each system. Coders were instructed to follow guidelines from the original published documents outlining system classification criteria for IFIC [17] and UNC [13]. In the case of Nova, multiple versions of the system have been published [14,29,30,31,32]. For this analysis, we used the criteria described in a 2014 review of classification systems by the authors of Nova [29], which has been referenced in subsequent publications by the authors. Published studies employing the systems were used to clarify application of the processing system [15,19,33,34]. NHANES food descriptors (Appendix A) associated with unique food codes were used in classifying foods. For mixed dished (e.g., pizza) foods were assumed to be homemade unless the food descriptor included place of production/production method (e.g., fast-food restaurant). In cases of ambiguity, coders were instructed to choose the more conservative processing category (i.e., less processed). For the IFIC and Nova systems, foods are classified into five and four categories, respectively, as presented in Table 1 [17,29]. The UNC system utilizes the same scheme as Nova, but further subdivides foods into seven processing categories (unprocessed/minimally, basic—preservation, basic—ingredient, moderately—grain product, moderately—flavor, highly—ingredient and highly) [13]. To examine inter-rater reliability, original processing category assignment was compared between coders (category 1–5 for IFIC; category 1–4 for Nova and category 1–7 for UNC). 

**Figure 1:**
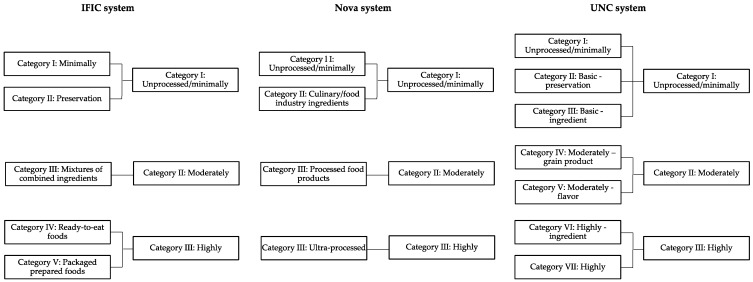
Original and final category assignment for foods as classified by the International Food Information Council (IFIC), Nova, and University of North Carolina at Chapel Hill (UNC) systems.

A third coder (author 1) evaluated coding discrepancies and determined a final coding decision by consultation with authors 3 and 5 for use in analyses examining the relationship between nutrient concentration and processing category. In order to compare systems on a common scale, processing classifications were collapsed to four categories: for IFIC, categories four (ready-to-eat processed) and five (foods/meals) were combined into category four. For UNC, categories two (basic—preservation) and three (basic—ingredient) were combined into category two; categories four (moderately—grain product) and five (moderately—flavor) were combined into category three, and categories six (highly—ingredient) and seven (highly) were combined into category four. Due to insufficient numbers of category two foods, categories one and two were combined for all systems. Other studies have found small proportions of foods classified as basic/processed for preservation compared to other categories, supporting the decision to combine this category with category one [15,34,35]. The resulting categories were category one (unprocessed/minimally), category two (moderately processed), and category three (highly processed) (Figure 1). 

### 2.4. Analysis 

All statistical analyses were performed using Stata version 15 (StataCorp; College Station, TX, USA) and R (R Core Team; Vienna, Austria; 2013). 

#### 2.4.1. Inter-Rater Reliability

For objective 1, Spearman’s rank correlation coefficient (*ρ*) was used to quantify inter-rater reliability of processing category assignment between coders. 

#### 2.4.2. Processing System Agreement 

For objective 2, we compared agreement between systems using Cohen’s kappa coefficient. Because the outcome of interest was agreement between processing systems, four-category processing ratings were used for comparison. Agreement was defined according guidelines published by Landis and Koch, where 0.00–0.20 is slight agreement, 0.21–0.40 is fair, 0.41–0.60 is moderate, 0.61–0.80 is substantial, and 0.81–1.00 is almost perfect [36]. 

#### 2.4.3. Relationship between Processing Category and Nutrient Concentration 

For objective 3, we used two analysis methods to investigate the relationship between processing classification and nutrient concentration. First, discriminant function analysis was used to allow for visual exploration of the ability of nutrient concentration to predict the processing category of foods as specified by each system. Next, the relationship was described further using logistic regression. A test of proportional odds was run to determine whether the assumptions of ordinal logistic regression were upheld. Results of this test indicated that multinomial logistic regression was preferable.

Both linear discriminant analysis and multinomial logistic regression are multivariate and provide information on individual dimensions, but offer different insight through post-estimation commands and visualization. We considered *p* values of less than 0.05 to be statistically significant.

## 3. Results

There was a total of 8661 children between six and twelve years old with two days of dietary recall in the 2013–2014 NHANES dataset. Among these participants, 5532 unique foods were reported during dietary recalls. The top five most commonly consumed foods by servings per day were 2% reduced fat milk, white bread, “tomato catsup” (ketchup), American cheese, and whole milk. The majority of the top 100 most commonly consumed foods were classified as highly processed, regardless of classification system used. However, the Nova system classified the most foods as highly processed (70%) compared to the UNC (62%) and IFIC (53%) systems. Appendix A lists all foods and processing category assignment, including common and discrepant classifications between systems. 

### 3.1. Inter-Rater Reliability 

Agreement between coders for processing category within classification systems, as measured by Spearman’s rank correlation coefficient (*ρ*), was high for the UNC system (*ρ* = 0.97, *p* < 0.001), and lower for the IFIC (*ρ* = 0.78, *p* < 0.001) and Nova (*ρ* = 0.76, *p* < 0.001) systems. 

### 3.2. Processing System Agreement 

Overall agreement between classification systems as measured by kappa statistic was moderate for all comparisons (0.41< kappa > 0.60). The Nova and IFIC systems had 70.0% agreement (expected agreement = 42.1; kappa = 0.48; *p* < 0.0001). Agreement was slightly higher for the Nova and UNC systems at 76.0% (expected agreement = 47.3; kappa = 0.54; *p* < 0.0001) and the IFIC and UNC systems at 75.0% (expected agreement = 39.2; kappa = 0.59; *p* < 0.0001). See Appendix A for commonly classified foods and disagreements between systems.

### 3.3. Relationship between Processing Category and Nutrient Concentration 

Mean nutrient concentrations by processing category for the IFIC, Nova, and UNC systems are presented in Table 2. Of the nutrients to discourage, added sugars and sodium had the lowest mean concentrations among category 1 foods and highest mean concentrations among category 3 foods for all three systems. Mean concentrations of nutrients to encourage by processing category were inconsistent among the three systems.

**Figure 2:**
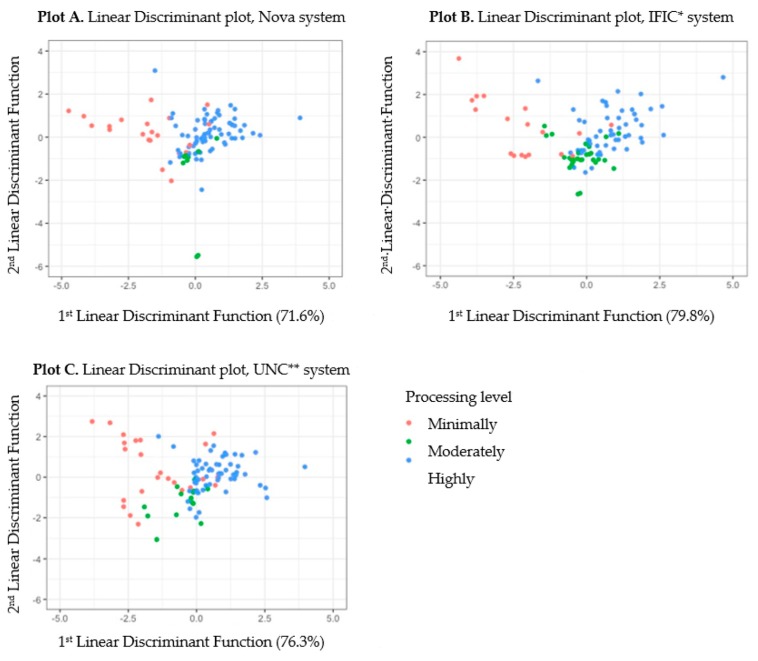
(**A**). Linear Discriminant plot, Nova system. (**B**). Linear Discriminant plot, IFIC * system. (**C**). Linear Discriminant plot, UNC ** system. The top 100 foods consumed by children 6–12 years old (NHANES 2013–2014) by processing category, plotted within the first two linear discriminants for the * International Food Information Council (IFIC), Nova and ** University of North Carolina at Chapel Hill (UNC) systems according to predicted classification category. Percentage of variance explained by the first and second linear discriminant is included in each figure.

Figure 2 depicts the relationships between the ability of nutrient concentration to differentiate between processing category for each system among the top 100 foods. The scatterplots display foods as defined by the linear discriminate functions when nutrient concentrations are considered for discrimination. Moderately processed foods are not well distinguished from minimally and highly processed foods by nutrient concentration amongst the three systems, demonstrated by considerable overlap between observations classified as moderately processed and other processing categories. For the IFIC system, 77.8% of minimally processed foods, 48.3% of moderately processed foods and 90.6% of highly processed foods were classified as predicted by the linear discriminant analysis. The first linear discriminate explained 79.8% of the between-group variance, and the second discriminate explained 20.2%. For the Nova system, 61.9% of minimally processed foods, 33.3% of moderately processed foods, and 95.7% of highly processed foods were classified as predicted by the linear discriminant analysis; the first discriminant function explained 71.6% of variability. For the UNC system, 54.2% of minimally processed foods, 57.1% of moderately processed foods, and 90.3% of highly processed foods were classified as predicted by the linear discriminant analysis. The first discriminant function explained 76.3% of variability for the UNC system. 

As seen in Figure 2, moderately processed foods are not well distinguished from minimally and highly processed foods by nutrient concentration amongst the three systems, demonstrated by considerable overlap between observations classified as moderately processed and other processing categories. The percent of variance described by the linear discriminant functions indicates how much discriminating power each function possesses. The first discriminant function for IFIC and UNC described a higher percentage of the variance (79.8% and 76.35, respectively) than Nova (71.6%). This suggests that processing categories as defined by IFIC and UNC are better aligned with nutrient concentration compared to Nova for the foods used in our analysis. 

The results of multinomial logistic regression models of the association between classification category and nutrients of concern among the top 100 foods are presented in Table 3. Of the overconsumed nutrients, higher added sugar was a significant predictor of moderately compared to minimally processed foods for the UNC system, and highly compared to minimally processed foods for all systems. Higher sodium was a significant predictor of moderately compared to minimally processed foods for Nova, and highly compared to minimally processed foods for all three systems. Of the under-consumed nutrients, lower potassium was a significant predictor of moderately processed compared to minimally processed foods for the IFIC system (Odds ratio = 0.97, *p* = 0.01, 95% CI [0.94, 0.99]). Lower vitamin D was a significant predictor of highly compared to minimally processed foods for the UNC system (Odds ratio = 0.06, *p* = 0.04, 95% CI [0.00, 0.83]). Given that a high number of foods contained listed value of zero for several nutrients, we removed five nutrients from the model: choline, magnesium, and vitamins A, E, and C. As specified by the DGA, these nutrients are consumed in amounts below the estimated average requirement or adequate intake categories, but are not considered nutrients of public health concern because low intakes are not associated with health concerns [28]. 

## 4. Discussion

As use of the term “processed” increases among researchers and the general public, there is a need for a commonly accepted classification system and definitions to describe processing categories [37,38]. In the U.S., processed food consumption has been examined with regard to racial/ethnic disparities [16,34], dietary quality [33], obesity [39], body fat [22], and weight gain [40], while globally, researchers have examined associations with obesity [41,42], lipid profiles [43], metabolic syndrome [44], cancer risk [45], and mortality, among others. These investigations have reached disparate conclusions, which may depend on the processing classification system used in analyses. Understanding the effects of processed food consumption during childhood may be particularly important since eating preferences and behaviors are established during this period [46]. Using a nationally representative sample of foods commonly consumed by children in the U.S., this study empirically demonstrates the effect of processing classification system on conclusions regarding the relationship between processing and nutrient concentration, highlighting common and discordant aspects of these systems as well nutritional components that best align with processing category. To our knowledge, this is the first study to evaluate and compare processing frameworks using quantitative outcome measures, including inter-rater reliability and ability to predict nutrient concentration.

Reliability is a fundamental concern of research involving any type of systematic coding. A primary objective of this study was to compare inter-rater reliability across processing classification frameworks to assess the relative objectivity and rigor of each system. We hypothesized that the UNC system would demonstrate higher reliability due to the provision of an exhaustive list of foods categorized by processing category [13]. As theorized, we saw the highest inter-rater reliability between coders classifying foods with the UNC system. The complexity of industrially produced foods in the U.S. is such that without precise category definitions and mutually exclusive categories, the potential for misclassification is high. The Nova system, as well as conclusions stemming from use of this system, have prompted extensive criticism from the scientific community [33,38,47,48]. In particular, critics have cited the lack of rigorous definitions for processing categories [47], undefined cutoff values for food additives and nutrients (despite reference to “high” amounts in ultra-processed foods [30]), and coding methodologies that change over time [38]. Others have questioned the usefulness of the Nova framework given the availability of nutrient profiling systems, which have reproducible algorithms and greater specificity [49]. Although based on the Nova system, the UNC system was developed specifically to categorize foods available in U.S. supermarkets [13]. The supplemental table provided by the authors categorizes all bar-coded foods by food groups, reducing subjectivity [13]. This suggests that a comprehensive framework for classifying foods is necessary to avoid misclassification. 

We theorized that the Nova and UNC systems would have the highest agreement, as assessed by Spearman’s rho, since the UNC system was directly adapted from the Nova framework. As expected, the UNC and Nova systems had the highest agreement, but there was considerable discordance between all systems for moderately processed (category II) foods (Appendix A). In particular, flavored milks were designated as moderately processed by the IFIC and UNC systems, and highly processed by Nova; most condiments and sauces were designated as moderately processed by the IFIC system, and highly processed by the Nova and UNC systems. The Nova system classified the most foods as highly processed (70%), while the IFIC system classified only 53% into the highest processing category, suggesting that the IFIC system underestimates the contributions of highly processed foods compared to Nova and UNC. 

Results of the multinomial logistic regression and linear discriminant analyses support the observation that all systems performed best when classifying highly processed foods; in other words, processing classification was most successful in distinguishing highly processed from minimally or moderately processed foods by nutrient profile. Higher added sugar and sodium categories were significant predictors of highly processed compared to minimally processed foods across all systems; this is not surprising, since the addition of flavorings (which likely include sugar and sodium) are criteria for higher processing as described in Table 1. This suggests that overconsumed nutrients may be better aligned with processing classification than under-consumed nutrients.

We found considerable overlap between foods classified as moderately processed with minimally and highly processed foods when plotted according to their linear discriminants in Figure 2. These plots indicate that nutrient concentrations were not strong predictors of processing category for the three systems. However, the first discriminant function for IFIC and UNC described a higher percentage of the variance (79.8% and 76.35, respectively) than Nova (71.6%). This suggests that processing categories as defined by IFIC and UNC are better aligned with nutrient concentration compared to Nova for the foods used in our analysis. 

From the top 100 foods used in the present analysis, it is possible to look at specific foods that are predictive of nutrient concentration by processing category, and ones that are not. Foods such as nonfat milk and fresh fruits and vegetables are high in under-consumed nutrients, and low in overconsumed nutrients. Granulated sugar is also a less or “basic” processed food; however, its nutrient profile is better aligned with those of highly processed foods. Similarly, plain peanut-butter and commercially prepared salsa, classified as highly-processed by two of three systems, are each high in several under-consumed nutrients. Research considering only the effects of processing category, without examining the specific nutrient concentrations of foods commonly consumed by children, may misclassify the healthfulness of certain foods. 

There is a limited research examining the relationship between processed food consumption and dietary quality in children. A 2005 study empirically investigated whether the increased prevalence of childhood obesity was associated with increased processed food consumption, finding that dietary energy density and food additives from the most processed foods may be a contributing factor [39]. This analysis defined processing by examining energy-dense foods and food residuals (non-nutritive food additives), preventing direct comparison with the present study. Our analysis did find that moderately processed foods are significantly more energy dense than minimally processed foods using the UNC system. Although we did not find significant effects for highly processed compared to less processed foods, there is a parallel increase in energy content with processing category for all systems, supporting the assumption that energy content increases with processing category (Table 2). Using reported foods from dietary records and 24 h recalls, an analysis of ultra-processed food consumption and dietary quality in children from Colombia (using the Nova system) found that highly-processed foods had greater concentrations of sugar, sodium, and trans-fatty acids, as well as lower categories of polyunsaturated fatty acids, vitamins A, B12, C, and E, calcium, and zinc [50]. The authors also found high categories of folate and iron in highly processed foods due to fortification. 

Although these results cannot be directly translated to the U.S. food system, our findings support the conclusion that processing systems are better aligned with overconsumed nutrients. Through extensive food fortification, highly processed foods may appear more similar to minimally and moderately processed foods with respect to under-consumed nutrients. In contrast, a study using the IFIC system examined children participating in NHANES 2003–2008 concluded that food from all processing categories contributed both under- and overconsumed nutrients as defined by the DGA [16]. Results do demonstrate that less processed foods contributed proportionally lower amounts of added sugars, sodium and energy, and higher amounts of several under-consumed nutrients. The authors point out that less processed foods are also higher in cholesterol; however, cholesterol is no longer considered a nutrient of concern according to the DGA. 

This study has several notable strengths and limitations. As the first study to compare processing coding of foods commonly consumed by children between classification systems, we chose to limit our investigation to the top 100 most commonly consumed foods by children. These foods do not represent a comprehensive view of total diet. However, we used NHANES 2013–2014, a large, well-designed national survey that is representative of children in the U.S., which also utilizes the gold standard approach for dietary data collection [23]. In examining the relationship between processing classification and nutrient concentration, we used a standardized 100 g portion for all foods; this amount is not necessarily reflective of what children actually eat, and may underestimate the role of processing category in determining nutrient concentrations in overall diets for foods eaten in large quantities (such as beverages), or overestimate the role for foods eaten in smaller quantities (such as condiments). However, the top 100 most commonly consumed foods were chosen based on reported consumption of standard servings, suggesting that the foods are reflective of consumption in the general child population. By collapsing processing categories into three categories, we may miss pertinent differences between minimally and basic processed foods. However, very small samples of moderately processed (category two) foods prevented independent analysis, suggesting that future work may benefit from closer examination of the role of these foods in children’s diets. The use of linear discriminant analysis in addition to multinomial logistic regression in the present study highlights key differences between systems, while highlighting potential strengths of processing classification with respect to dietary quality assessment; this methodology could be applied to larger food samples and populations to explore similar research questions.

## 5. Conclusions

The impact of processing classification systems on conclusions regarding the relationship between processing category and nutrient content is significant. Without established definitions of processing categories and a rigorous framework to guide food coding, misclassifications will persist. Current processing systems may be better aligned with overconsumed dietary components, including added sugars and sodium, with our results highlighting the lack of ability of three processing systems to distinguish moderately processed foods from minimally or highly processed ones by nutrient concentration. In considering recommendations for children’s diets, establishment of a nationally recognized processing framework for the U.S. food system should consider categorizations that align with nutrient content to increase utility. 

## Figures and Tables

**Table 1:** Category definitions and criteria for classifying foods and beverages based on degree of industrial food processing according to the IFIC ^a^ [15], Nova [14], and UNC ^b^ [13] systems.

	Category I	Category II	Category III	Category IV	Category V	Category VI	Category VII
IFIC	Minimally processed: Foods that require little processing or production, which retain most of their inherent properties.	Foods processed for preservation: Foods processed to help preserve and enhance nutrients and freshness of foods at their peak.	Mixtures of combined ingredients; Foods containing sweeteners, spices, oils, colors, flavors, and preservatives used for promotion of safety, taste, visual appeal.	Ready-to-eat processed: Foods needing minimal or no preparation. Group subdivided into ‘packaged ready-to-eat foods’ and ‘mixtures possibly store prepared.’	Prepared foods/meals: Foods packaged for freshness and ease of preparation.	NA	NA
	Examples: Milk, coffee, fruit, vegetables, meat and eggs.	Fruit juices; cooked, canned, or frozen vegetables and fruits.	Breads or rolls; sugars and sweeteners, cheeses, various condiments, and tacos or tortillas.	Soft drinks, sweets, salty snacks, cereal, lunchmeats, and alcoholic beverages.	Pizza, prepared meat dishes, pasta, and prepared meals.	NA	NA
Nova	Unprocessed & minimally processed: Foods of plant origin or animal origin, shortly after harvesting, gathering, slaughter or husbanding; foods altered in ways that do not add or introduce any substance	Processed culinary ingredients: Food products extracted and purified by industry from constituents of foods, or else obtained from nature, such as salt.	Processed foods: Manufactured by adding substances like oil, sugar or salt to whole foods, to make them durable and more palatable and attractive.	Ultra-processed foods: Formulated mostly or entirely from substances derived from foods. Processes include hydrogenation, hydrolysis; extruding, molding, reshaping; preprocessing by frying, baking.	NA	NA	NA
	Examples: Fresh or frozen vegetables and fruits; grains including all types of rice; freshly prepared or pasteurized non-reconstituted fruit juices; fresh, dried, frozen meats; dried, fresh, pasteurized milk.	Plant oils; animal fats; sugars and syrups; starches and flours, uncooked ‘raw’ pastas made from flour and water, salt.	Canned or bottled vegetables in brine; fruits preserved in syrup; tinned whole or pieces of fish preserved in oil; salted nuts; un-reconstituted processed meat and fish such as ham, bacon, smoked fish; cheese.	Confectionery; burgers and hot dogs; breaded meats; breads, buns, cookies (biscuits); breakfast cereals; ‘energy’ bars; sauces; cola, ‘energy’ drinks; sweetened yoghurts; fruit and fruit ‘nectar’ drinks; pre-prepared dishes.	NA	NA	NA
UNC	Unprocessed & minimally processed: Single-ingredient foods with no or very slight modifications that do not change inherent properties of the food as found in its natural form.	Processed basic ingredients: single isolated food components obtained by extraction or purification using physical or chemical processes that change inherent properties of the food.	Processed for basic preservation or precooking: single minimally processed foods modified by physical or chemical processes for the purpose of preservation or precooking but remaining as single foods.	Moderately processed for flavor: single minimally or moderately processed foods with addition of flavor additives for the purpose of enhancing flavor	Moderately processed grain products: grain products made from whole-grain flour with water, salt, and/or yeast.	Highly processed ingredients: multi-ingredient industrially formulated mixtures processed to the extent that they are no longer recognizable as their original plant/animal source.	Highly processed stand-alone: multi-ingredient industrially formulated mixtures processed to the extent that they are no longer recognizable as their original plant/animal source.
	Examples: Plain milk; fresh, frozen or dried plain fruit or vegetables; eggs, unseasoned meat; whole grain flour and pasta; brown rice; honey, herbs and spices.	Unsweetened fruit juice not from concentrate; whole grain pasta; oil, unsalted butter, sugar, salt.	Unsweetened fruit juice from concentrate; unsweetened/unflavored canned fruit, vegetables, legumes; plain peanut butter, refined grain pasta, white rice; plain yogurt.	Sweetened fruit juice, flavored milk; frozen French fries; salted peanut butter; smoked or cure meats; cheese, flavored yogurt, salted butter.	Whole grain breads, tortillas or crackers with no added sugar or fat.	Tomato sauce, salsa, mayonnaise, salad dressing, ketchup.	Soda, fruit drinks; formed lunchmeats; breads made with refined flours; pastries; ice-cream, processed cheese; candy.

^a^ International Food Information Council; ^b^ University of North Carolina at Chapel Hill.

**Table 2:** Mean nutrient concentration per 100 g of the top 100 foods consumed by children 6–12 years old by processing category for the IFIC ^a^ [15], Nova [14], and UNC ^b^ [13] systems, NHANES ^c^ 2013–2014.

	IFIC						Nova						UNC					
	Processing Category (*n*) ^d^																	
Nutrient, Mean (sd)	Category 1 (18)		Category 2 (29)		Category 3 (52)		Category 1 (21)		Category 2 (9)		Category 3 (70)		Category 1 (24)		Category 2 (14)		Category 3 (62)	
Energy (kcal)	91.2	(44.8)	107.2	(85.7)	151.8	(88.6)	98.6	(55.3)	74.1	(27.9)	143.7	(92.2)	103.0	(54.2)	93.0	(59.1)	145.5	(95.0)
Added sugars (g)	1.7	(6.3)	3.2	(5.6)	9.8	(10.9)	1.8	(5.6)	2.5	(7.0)	8.4	(10.2)	2.2	(6.8)	5.6	(7.9)	8.3	(10.3)
Sat fat (g)	0.8	(1.5)	1.6	(2.1)	1.7	(2.1)	1.1	(2.0)	1.9	(1.1)	1.6	(2.1)	0.9	(1.4)	2.0	(2.1)	1.7	(2.2)
Sodium (mg)	46.1	(69.8)	167.4	(108.2)	223.3	(229.5)	64.2	(98.7)	160.2	(80.0)	210.4	(207.8)	88.2	(120.5)	119.5	(72.6)	221.4	(214.8)
Fiber (g)	1.2	(1.5)	0.5	(0.7)	0.9	(1.1)	1.0	(1.4)	0.0	(0.0)	0.9	(1.0)	1.0	(1.3)	0.5	(0.8)	0.9	(1.1)
Potassium (mg)	253.8	(132.4)	80.2	(120.7)	110.3	(131.0)	218.7	(150.7)	46.6	(40.5)	110.4	(134.3)	205.3	(143.4)	151.2	(171.0)	91.9	(120.2)
Choline (mg)	16.6	(16.7)	17.6	(32.4)	10.8	(13.8)	14.5	(16.3)	32.2	(52.8)	11.3	(14.2)	23.5	(34.6)	14.7	(16.4)	9.9	(13.2)
Magnesium (mg)	16.3	(9.5)	10.1	(11.0)	13.8	(11.6)	14.9	(10.1)	4.5	(2.0)	13.8	(11.8)	15.7	(9.4)	13.7	(12.6)	12.2	(11.5)
Calcium (mg)	79.9	(117.9)	66.6	(87.5)	52.0	(87.2)	69.1	(112.0)	48.2	(52.5)	60.6	(91.8)	65.0	(104.9)	113.7	(121.4)	48.0	(77.1)
Iron (mg)	0.3	(0.4)	0.6	(0.6)	1.7	(2.6)	0.4	(0.5)	0.3	(0.4)	1.5	(2.3)	0.5	(0.6)	0.3	(0.3)	1.6	(2.4)
Vit A (mcg RAE)	94.1	(249.5)	35.0	(50.3)	47.7	(89.0)	85.3	(231.7)	34.8	(34.2)	44.7	(82.5)	77.5	(217.4)	44.7	(51.7)	44.3	(85.0)
Vit D (mcg)	0.7	(1.3)	0.5	(0.9)	0.3	(0.6)	0.6	(1.2)	0.4	(0.5)	0.4	(0.8)	0.6	(1.1)	0.9	(1.3)	0.2	(0.5)
Vit E (mg)	0.2	(0.2)	0.3	(0.4)	0.6	(0.7)	0.2	(0.2)	0.3	(0.4)	0.5	(0.7)	0.3	(0.4)	0.1	(0.1)	0.5	(0.7)
Vit C (mg)	21.0	(31.6)	0.2	(0.5)	3.0	(5.5)	18.0	(30.1)	0.0	(0.0)	2.3	(4.9)	15.7	(28.7)	0.5	(1.0)	2.5	(5.1)

^a^ International Food Information Council; ^b^ University of North Carolina at Chapel Hill; ^c^ National Health and Nutrition Examination Survey; ^d^ Processing classifications collapsed to three categories for analysis: for IFIC, categories four (ready-to-eat processed) and five (prepared foods/meals) were combined into category four. For UNC, categories two (basic—preservation) and three (basic—ingredient) were combined into category two; categories four (moderately—grain product) and five (moderately—flavor) were combined into category three, and categories six (highly—ingredient) and seven (highly) were combined into category four. Due to insufficient numbers of category two foods, categories one and two were combined for all systems.

**Table 3:** Multinomial logistic regression results for the association between classification category as determined by the IFIC ^a,15^, Nova ^14^, and UNC ^b,13^ processing classification systems and concentration of nutrients of concern ^c^ among the top 100 foods most commonly consumed among 6–12-year-olds in the U.S. population ^d^, NHANES ^e^ 2013–2014.

	IFIC			Nova			UNC		
Nutrient, Mean (sd) *	OR **	95% CI	*p*	OR	95% CI	*p*	OR	95% CI	*p*
Category 1 (base outcome, minimally processed) ^f^									
Category 2 (moderately processed)									
Energy (kcal)	1.01	(0.98, 1.05)	0.44	0.98	(0.93, 1.04)	0.58	0.96	(0.93, 1.00)	0.03
Added sugars (g)	1.05	(0.87, 1.28)	0.58	1.19	(0.89, 1.58)	0.24	1.25	(1.05, 1.49)	0.01
Sodium (mg)	1.03	(1.00, 1.07)	0.03	1.04	(1.01, 1.08)	0.02	1.01	(1.00, 1.02)	0.10
Saturated fat (g)	0.65	(0.28, 1.53)	0.32	1.09	(0.41, 2.93)	0.86	3.33	(1.21, 9.16)	0.02
Fiber (g)	3.10	(0.81, 11.83)	0.10	0.00		0.99	1.69	(0.44, 6.48)	0.45
Potassium (mg)	0.97	(0.94, 0.99)	0.01	0.95	(0.89, 1.01)	0.08	1.00	(0.99, 1.01)	0.87
Calcium (mg)	1.02	(0.98, 1.06	0.40	1.01	(0.98, 1.04)	0.63	1.02	(0.99, 1.05)	0.12
Iron (mg)	0.32	(0.08, 1.35)	0.12	24.18	(0.03, 20875.62)	0.36	0.89	(0.20, 4.03)	0.88
Vit D (mcg)	2.00	(0.05, 88.05)	0.72	0.54	(0.00, 66.41)	0.80	0.18	(0.01, 2.93)	0.23
Category 3 (highly processed)									
Energy (kcal)	1.00	(0.97, 1.04)	0.86	0.99	(0.97, 1.01)	0.54	0.99	(0.97, 1.01)	0.16
Added sugars (g)	1.25	(1.03, 1.51)	0.02	1.21	(1.05, 1.39)	0.01	1.23	(1.07, 1.40)	0.00
Sodium (mg)	1.04	(1.01, 1.07)	0.02	1.02	(1.00, 1.03)	0.01	1.01	(1.00, 1.02)	0.02
Fiber (g)	1.98	(0.56, 6.98)	0.29	1.51	(0.66, 3.44)	0.32	1.43	(0.58, 3.54)	0.44
Saturated fat (g)	1.01	(0.44, 2.31)	0.98	0.98	(0.57, 1.71)	0.96	2.18	(0.85, 5.55)	0.10
Potassium (mg)	0.99	(0.97, 1.00)	0.11	0.99	(0.99, 1.00)	0.22	1.00	(0.99, 1.00)	0.42
Calcium (mg)	1.01	(0.97, 1.06)	0.57	1.01	(0.98, 1.03)	0.52	1.01	(0.99, 1.04)	0.22
Iron (mg)	1.04	(0.38, 2.83)	0.94	1.11	(0.54, 2.27)	0.78	1.57	(0.81, 3.07)	0.19
Vit D (mcg)	0.16	(0.00, 6.82)	0.34	0.46	(0.04, 5.18)	0.53	0.06	(0.00, 0.83)	0.04

^a^ International Food Information Council; ^b^ University of North Carolina at Chapel Hill; ^c^ Concentration of nutrients per 100 grams; ^d^ Calculated according to servings consumed per day; ^e^ National Health and Nutrition Examination Survey; ^f^ Processing classifications collapsed to three categories for analysis: for IFIC, categories four (ready-to-eat processed) and five (prepared foods/meals) were combined into category four. For UNC, categories two (basic—preservation) and three (basic—ingredient) were combined into category two; categories four (moderately—grain product) and five (moderately—flavor) were combined into category three, and categories six (highly—ingredient) and seven (highly) were combined into category four. Due to insufficient numbers of category two foods, categories one and two were combined for all systems. * Nutrient concentrations calculated for 100 g of each food; ** Odds Ratio.

## Appendix A

**Table A1:** Corresponding and discrepant processing category assignments among the IFIC, Nova, and UNC classification systems for the top 100 most commonly consumed foods among children 6–12 years old (NHANES 2013–2014).

**Unprocessed/Minimally Unprocessed/Minimally Processed**			
**All systems**	**Classification unique to IFIC**	**Classification unique to Nova**	**Classification unique to UNC**
Milk, reduced fat (2%)	Tea, iced, bottled, black		Tea, iced, bottled, black
Milk, whole		Rice, white, cooked, fat added in cooking, made with oil	Rice, white, cooked, fat added in cooking, made with oil
Apple, raw		Butter, stick, salted	
Milk, low fat (1%)		Maple and corn and/or cane pancake syrup blends (formerly Corn and maple syrup (2% maple))	
Banana, raw		Pancake syrup, NFS	
Apple juice, 100%			Rice, white, cooked, fat not added in cooking
Lettuce, raw			Egg, whole, fried with oil
Orange juice, 100%, canned, bottled or in a carton			Egg omelet or scrambled egg, made with oil
Sugar, white, granulated or lump			Peanut butter
Orange, raw			Fruit flavored smoothie drink, frozen (no dairy)
Milk, fat free (skim)			
Fruit juice blend, 100% juice			
Grapes, raw, NS as to type			
Watermelon, raw			
Ground beef or patty, cooked, NS as to percent lean			
Strawberries, raw			
Carrots, raw			
**Moderately Processed**			
**All systems**	**Classification unique to IFIC**	**Classification unique to Nova**	**Classification unique to UNC**
Cheese, Cheddar	Egg omelet or scrambled egg, made with oil	Egg omelet or scrambled egg, made with oil	
Cheese, Mozzarella, part skim	Egg, whole, fried with oil	Egg, whole, fried with oil	
Queso Fresco	Bread, wheat or cracked wheat		Bread, wheat or cracked wheat
	Tortilla, corn		Tortilla, corn
		Pork bacon, NS as to fresh, smoked or cured, cooked	Pork bacon, NS as to fresh, smoked or cured, cooked
	Chocolate milk, ready to drink, reduced fat (2%)		Chocolate milk, ready to drink, reduced fat (2%)
	Strawberry milk, NFS		Strawberry milk, NFS
	Chocolate milk, ready to drink, low fat (1%)		Chocolate milk, ready to drink, low fat (1%)
	Chocolate milk, ready to drink, whole		Chocolate milk, ready to drink, whole
	Butter, stick, salted		Butter, stick, salted
	Italian dressing, made with vinegar and oil	Italian dressing, made with vinegar and oil	
	Rice, white, cooked, fat not added in cooking		
	Rice, white, cooked, fat added in cooking, made with oil		
	Bread, white		
	Tomato catsup		
	Creamy dressing		
	Pancakes, plain		
	Waffle, plain		
	Salsa, red, commercially-prepared		
	Roll, white, hamburger bun		
	Mustard		
	Mayonnaise, regular		
	Maple and corn and/or cane pancake syrup blends (formerly Corn and maple syrup (2% maple))		
	Roll, white, soft		
	Macaroni or noodles with cheese, made from packaged mix		
	Pancake syrup, NFS		
	Barbecue sauce		
		Fruit flavored smoothie drink, frozen (no dairy)	
		Pork sausage	
			Ham, sliced, prepackaged or deli, luncheon meat
			Yogurt, fruit, low fat milk
			Tea, iced, brewed, black, pre-sweetened with sugar
**Highly Processed**			
**All systems**	**Classification unique to IFIC**	**Classification unique to Nova**	**Classification unique to UNC**
Cheese, American	Peanut butter	Peanut butter	
Ice cream, regular, flavors other than chocolate	Ham, sliced, prepackaged or deli, luncheon meat	Ham, sliced, prepackaged or deli, luncheon meat	
Salty snacks, corn or cornmeal base, tortilla chips		Bread, white	Bread, white
Soft drink, fruit flavored, caffeine free		Tomato catsup	Tomato catsup
Cookie, chocolate chip		Creamy dressing	Creamy dressing
Chicken or turkey loaf, prepackaged or deli, luncheon meat		Waffle, plain	Waffle, plain
White potato chips, regular cut		Salsa, red, commercially-prepared	Salsa, red, commercially-prepared
Gatorade G sports drink		Roll, white, hamburger bun	Roll, white, hamburger bun
Pizza with pepperoni, from restaurant or fast food, regular crust		Mustard	Mustard
Fruit flavored drink, powdered, reconstituted		Mayonnaise, regular	Mayonnaise, regular
Soft drink, cola		Roll, white, soft	Roll, white, soft
Salty snacks, corn or cornmeal base, corn puffs and twists; corn-cheese puffs and twists		Macaroni or noodles with cheese, made from packaged mix	Macaroni or noodles with cheese, made from packaged mix
Capri Sun, fruit juice drink		Barbecue sauce	Barbecue sauce
Chicken nuggets, from fast food/restaurant	Pork sausage		Pork sausage
Soup, mostly noodles	Tea, iced, brewed, black, pre-sweetened with sugar		
Fruit leather and fruit snacks candy	Yogurt, fruit, low fat milk		
Jelly, all flavors	Fruit flavored smoothie drink, frozen (no dairy)		
Cracker, snack	Pork bacon, NS as to fresh, smoked or cured, cooked		
Pizza, cheese, from restaurant or fast food, regular crust		Tea, iced, brewed, black, pre-sweetened with sugar	
White potato, French fries, from fast food/restaurant		Yogurt, fruit, low fat milk	
Ice pop		Tea, iced, bottled, black	
Breakfast tart		Rice, white, cooked, fat not added in cooking	
Pretzels, hard		Bread, wheat or cracked wheat	
Soft taco with meat		Tortilla, corn	
Spaghetti with tomato sauce and meatballs or spaghetti with meat sauce or spaghetti with meat sauce and meatballs		Chocolate milk, ready to drink, reduced fat (2%)	
Froot Loops		Strawberry milk, NFS	
Quesadilla, just cheese, meatless		Chocolate milk, ready to drink, low fat (1%)	
Cookie, butter or sugar		Chocolate milk, ready to drink, whole	
Frosted Flakes, Kellogg's		Pancakes, plain	
Cake or cupcake, chocolate, devil's food or fudge, with icing or filling			Pancake syrup, NFS
Lemonade, fruit flavored drink			Italian dressing, made with vinegar and oil
Cheese, cream			Maple and corn and/or cane pancake syrup blends (formerly Corn and maple syrup (2% maple))
Cookie, animal			
Honey Nut Cheerios			
Soft drink, pepper type			
Hard candy			
Cookie, chocolate sandwich			
Corn dog (frankfurter or hot dog with cornbread coating)			
Cheerios			
Chicken patty, fillet, or tenders, breaded, cooked			
Cinnamon Toast Crunch			
Lucky Charms			
French toast, plain			
Bread, garlic			
Ice cream, regular, chocolate			
